# Marital Status and Survival in Pancreatic Cancer Patients: A SEER Based Analysis

**DOI:** 10.1371/journal.pone.0021052

**Published:** 2011-06-15

**Authors:** Michael Baine, Freshta Sahak, Chi Lin, Subhankar Chakraborty, Elizabeth Lyden, Surinder K. Batra

**Affiliations:** 1 Eppley Institute for Research in Cancer and Allied Diseases, University of Nebraska Medical Center, Omaha, Nebraska, United States of America; 2 Department of Biochemistry and Molecular Biology, University of Nebraska Medical Center, Omaha, Nebraska, United States of America; 3 Department of Radiation Oncology, University of Nebraska Medical Center, Omaha, Nebraska, United States of America; 4 College of Public Health, University of Nebraska Medical Center, Omaha, Nebraska, United States of America; Technische Universität München, Germany

## Abstract

**Background:**

Recent findings suggest that marital status affects survival in patients with different types of cancer. However, its role in the survival of patients with pancreatic ductal adenocarcinoma is unknown. In this study, we investigated whether there was an association between marital status and overall survival (OS) in patients with pancreatic ductal adenocarcinoma (PDAC).

**Methods:**

Adult patients diagnosed with PDAC between 1998 and 2003 with known marital statuses were identified from the Surveillance, Epidemiology, and End Results registry of the National Cancer Institute. OS for these patients was plotted using the Kaplan-Meier method. Comparative risks of mortality were evaluated by using univariate and multivariate-adjusted Cox regression models.

**Results:**

Using Kaplan-Meier analysis, we found that the median overall survival of patients was 4 months and 3 months (p<0.001) for married and unmarried patients, respectively. Subgroup analysis on patients with cancer-directed surgery showed that the median survival was 16 months and 13 months (P<0.0005) for married and unmarried groups, respectively. Multivariate analysis adjusting for age, race, sex, stage, year of diagnosis, radiation therapy and cancer-directed surgery showed that patients who were married at the time of diagnosis had a significantly decreased risk of death at both 2 months (15% risk reduction) and 3 years (13% risk reduction) post diagnosis.

**Conclusions:**

Marital status is an independent prognostic factor of both perioperative and long-term survival in patients with PDAC. This observation may suggest a suboptimally met psychosocial need among PDAC patients that is partially fulfilled by the support system provided by marriage.

## Introduction

42,470 individuals were diagnosed with pancreatic cancer and 32,300 individuals died from it in 2009, as estimated by The American Cancer Society [1]. Pancreatic cancer patients that have been reported to the Surveillance, Epidemiology, and End Results (SEER) database of the National Cancer Institute from 1996 to 2004 have an overall 5-year relative survival rate of 5%, making it the deadliest type of cancer [2]. Patients with pancreatic cancer rarely respond positively to treatment, thus making any pursuable avenue that may increase survival in these patients of significant importance.

It is well known that patient and environmental factors influence patient survival. It has been shown that a patient's overall mental state of well-being, including the desire to live and available social support has a significant effect on both their short and long term outcome. It has been shown that married persons receive better social support, including practical support and financial resources, decreasing the pressure of everyday stressors such as transportation, paperwork and household chores, so that the patient can focus on treatment. Marriage may also influence behaviors such as health screenings, diet, and exercise, all of which are factors that promote health and have been shown to prolong life [3].

Understanding how marriage influences survival will shed light on the importance of social support mechanisms in management of various diseases, including cancers. Marital status has been found to impact the survival of various types of cancer including kidney, bladder, prostate, breast, and colon cancers [4]. However, little is known about the relationship between marital status and survival in patients with pancreatic ductal adenocarcinoma (PDAC). An investigation into the relationship between marital status and the survival of patients with PDAC opens up a potential mechanism that may be influencing the survival of patients with the world's deadliest cancer, allowing for the establishment of more holistic approaches to PDAC treatment that may positively influence patient mortality.

## Materials and Methods

*Ethics statement:* This study has been reviewed by the UNMC Office of Regulatory Affairs (ORA) and has been determined to not be subject to review by the Institutional Review Board (IRB) under HHS or FDA regulations for the protection of human subjects.

Patients were identified from the SEER database (period 1988 to 2003) with age ≥18 years diagnosed with PDAC whose marital status was also known at the time of diagnosis. Patients with missing data on stage, external beam radiation therapy and cancer-directed surgery were excluded. Also excluded were patients who received non-external beam radiation therapy (EBRT).

**Figure 1:**
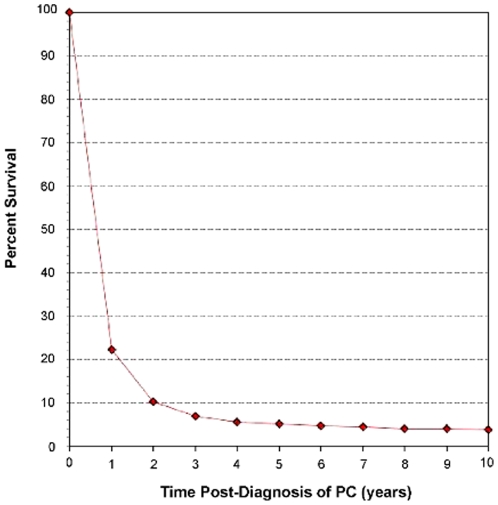
Relative Survival of Patients Diagnosed with Pancreatic Ductal Adenocarcinoma using SEER 9 data from 1988–2006. Survival of patients with pancreatic ductal adenocarcinoma (PC) can be separated into two qualitative phases. The first phase occurs in the initial 3 years post-PC diagnosis in which patient survival declines steeply. After 3 years post-diagnosis, PC patient survival declines much more slowly in what can be considered the second phase of the PC survival curve. The nature of these phases allows for 3-year survival to be considered a reasonable endpoint for evaluation of long-term PC patient survival. Annual survival estimates were calculated using monthly intervals.

Data were analyzed using the SAS 9.2 statistical software (Cary, NC, USA). Overall survival curves were generated using the Kaplan-Meier method and comparative risks of mortality were evaluated using univariate and multivariate Cox regression models [5]. Factors including marital status, sex, race, age at diagnosis, year of diagnosis, cancer-directed surgical status, EBRT status, and tumor stage were evaluated. Evaluation of tumor grade was excluded from this analysis since over half of the sample population had unknown grade. The SEER database defines cancer-directed surgery as partial or total pancreatectomy with or without regional lymph node dissection, subtotal gastrectomy, and duodenectomy, excluding incisional biopsy, exploratory surgery, bypass, any stand-alone surgery ending in the suffix –otomy or –ostomy, and any surgical procedures performed solely for diagnostic, staging, or palliative purposes (SEER Program code manual, 2009). Due to the fact that cancer-directed surgery is well known to have a significant impact on survival in patients with PDAC, multivariate analyses were repeated beyond the patient set as a whole to evaluate if the various parameters found to affect patient survival in PDAC patients remained significant prognostic factors of survival in sub-populations of patients who either did or did not undergo cancer-directed surgery. All parameters were evaluated at two time-points: 2 months post-diagnosis and 3 years post-diagnosis. The 2 month time point was chosen due to the fact that cancer-directed surgery for pancreatic adenocarcinoma is often performed within the first 6–10 weeks post-diagnosis and is associated with high morbidity which can itself lead to early mortality. As such, the 2 month time point, considered perioperative, represents an important time point in patient therapy as well as representing general short-term survival. The 3 year time point was chosen to represent long-term survival because the 3 year mark represents the end of the steep decline in the pancreatic adenocarcinoma survival curve, after which the slope of patient survival flattens (Figure 1), thus making 3 year survival an acceptable working definition of long-term survival in PDAC. For analysis of both 2 month and 3 year survivals, data for all patients with longer survival times were censored at the respective time points.

## Results

### Patient characteristics

There were 72,727 cases of pancreatic adenocarcinoma reported in the SEER database from 1973 to 2006. All cases in which diagnoses were made before 1988 (17287) were excluded because of incomplete information on staging and surgery. Cases diagnosed from 2004 to 2006 (15385) were also excluded to allow calculation of 3 year survival. We also removed cases with missing information on marriage (1264), surgery (342), external beam radiation therapy (EBRT) (729), stage (3157), and pediatric cases (8), ending up with a final sample of 34,555 cases (married: 20761, unmarried 13794).

Patient characteristics with different marital statuses are described in Table 1. Patients who were married at the time of diagnosis were more likely to be white, male and younger as compared to their unmarried counterparts. Additionally, married patients were more likely to have undergone cancer directed surgery as well as EBRT.

**Table 1:** Patient Characteristics.

	No. of patients (%)				
	Unmarried		Married		
Characteristic	(N = 13794)		(N = 20761)		P
Sex					<0.0001
Male	4792	(35)	12971	(62)	
Female	9002	(67)	7790	(38)	
Race					<0.0001
White	10756	(78)	17501	(84)	
Black	2238	(16)	1638	(8)	
Other	800	(6)	1622	(8)	
Age					
Median Age at Diagnosis	71		67		<0.0001
18–60 y/o	2894	(21)	5291	(25)	
60–74 y/o	5381	(39)	10126	(49)	
>74 y/o	5519	(40)	5344	(27)	
Year of Diagnosis					0.0384
2001–2003	4961	(36)	7224	(35)	
1998–2000	2959	(21)	4413	(21)	
1193–1997	3431	(25)	5237	(25)	
1988–1992	2443	(18)	3887	(19)	
Cancer Directed Surgery					<0.0001
None	12180	(88)	17557	(85)	
Surgery	1614	(12)	3204	(15)	
Radiation Therapy					<0.0001
None	11505	(83)	16067	(77)	
EBRT	2289	(17)	4694	(23)	
Stage					0.0007
Localized	1154	(8)	1507	(7)	
Regional	4157	(30)	6369	(31)	
Distant	8483	(61)	12885	(62)	

EBRT indicates external beam radiation therapy; A race of Other indicates Asian, or Asian-Pacific Islander.

### Overall survival 3-years post diagnosis by marriage

**Figure 2:**
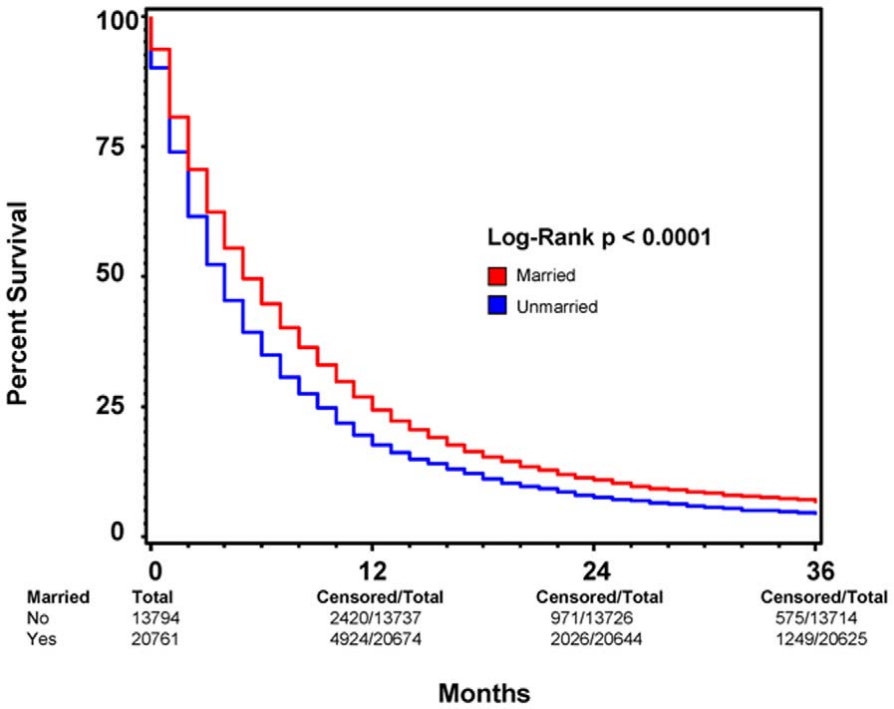
Kaplan-Meier Survival Curves Comparing Patient Marital Status. A Kaplan-Meier survival curve comparing survival based on marital status among all patients diagnosed with pancreatic ductal adenocarcinoma (PC) between 1998 and 2003 with known marital status shows increased survival among married patients as compared to those who were non-married at the time of diagnosis. RED indicates patients who were married at the time of diagnosis and BLUE represents patients who were not married at the time of diagnosis. Total patients for each patient group, as well as total and censored patients at 12, 24, and 36 months are indicated. For each time point, “total” indicates the number of patients with ample follow-up information to determine survival at the respective time point. “Censored” indicates the number of patients who lived beyond the respective time point. This method of data representation was chosen rather than indicating at-risk patients for each time point to allow for easier interpretation of overall survival at 12, 24, and 36 months. Total patient number indicated at 0 months is equivalent to total at-risk for each patient group. For each time point, “at-risk” can be considered the number of “censored” patients from the previous time point minus the number of patients lost to follow-up between the previous and current time points. Patients lost to follow-up between time points are indicated by the respective decrease in “total” values.

As shown in Figure 2, we plotted Kaplan-Meier curves and used the log-rank test to compare overall survival rate in the married group with that in the unmarried group. The median survival and 3-year overall survival were 5 (95% CI: 4.9–5.1) months and 6% respectively for the married group, compared with 4 (95% CI: 3–4) months and 4% for the unmarried group (p<0.001). Upon univariate analysis, factors that were associated with decreased 3-year overall survival in PDAC patients included male sex, non-Caucasian race, increased age at diagnosis, earlier year of diagnosis, lack of cancer-directed surgery, lack of radiation therapy, more advanced stage, and being unmarried. Multivariate analysis using the Cox proportional harzard model was performed to compare the married group with unmarried group after controlling for all above mentioned covariates. The results are reported in Table 2. Factors that continued to correlate with decreased 3-year overall survival included being male, African American race, increased age at diagnosis, earlier year of diagnosis, lack of cancer-directed surgery, lack of radiation therapy, higher stage, and being unmarried. Positive marital status resulted in a 13% decrease in risk of death at 3 years post diagnosis for patients with PDAC after adjusting for age, gender, race, year of diagnosis, stage, cancer-directed surgery and EBRT.

**Table 2:** Univariate and Multivariate Analysis of Overall 3 year Survival in Patients with Pancreatic Ductal Adenocarcinoma.

	Univariate analysis			Multivariate analysis		
Variable	HR (95%CI)		P	HR (95%CI)		P
Marital Status						
Unmarried	1.00			1.00		
Married	0.83	(0.82–0.85)	<0.0001	0.87	(0.85–0.89)	<0.0001
Sex						
Men	1.00			1.00		
Women	0.97	(0.95–0.99)	0.0100	0.91	(0.89–0.94)	<0.0001
Race						
White	1.00			1.00		
Black	1.12	(1.08–1.16)	<0.0001	1.12	(1.08–1.16)	<0.0001
Other* ≠	1.00	(0.96–1.04)	0.8869	0.99	(0.94–1.03)	0.4988
Age at Diagnosis						
<60	1.00			1.00		
60–74	1.28	(1.25–1.32)	<0.0001	1.30	(1.26–1.34)	<0.0001
>74	1.67	(1.62–1.72)	<0.0001	1.63	(1.58–1.68)	<0.0001
Year of Diagnosis						
2001–2003	1.00			1.00		
1998–2000	1.05	(1.02–1.08)	0.0014	1.05	(1.02–1.08)	0.001
1993–1997	1.12	(1.09–1.15)	<0.0001	1.13	(1.10–1.16)	<0.0001
1988–1992	1.17	(1.12–1.20)	<0.0001	1.19	(1.15–1.22)	<0.0001
Cancer Directed Surgery						
None	1.00			1.00		
Surgery	0.34	(0.32–0.35)	<0.0001	0.46	(0.4–0.48)	<0.0001
Radiation Therapy						
None	1.00			1.00		
EBRT	0.56	(0.54–0.57)	<0.0001	0.72	(0.70–0.74)	<0.0001
Stage						
Localized	1.00			1.00		
Regional	1.13	(1.08–1.18)	<0.0001	1.21	(1.16–1.27)	<0.0001
Distant	2.23	(2.13–2.33)	<0.0001	1.83	(1.75–1.91)	<0.0001

HR indicates hazard ratio; CI indicates confidence interval; EBRT, external beam radiation therapy; A race of Other indicates Asian, or Asian-Pacific Islander.

*Variable shown to not be predictive of survival in univariate analysis.

≠Variable shown to not be predictive of survival in multivariate analysis.

**Figure 3:**
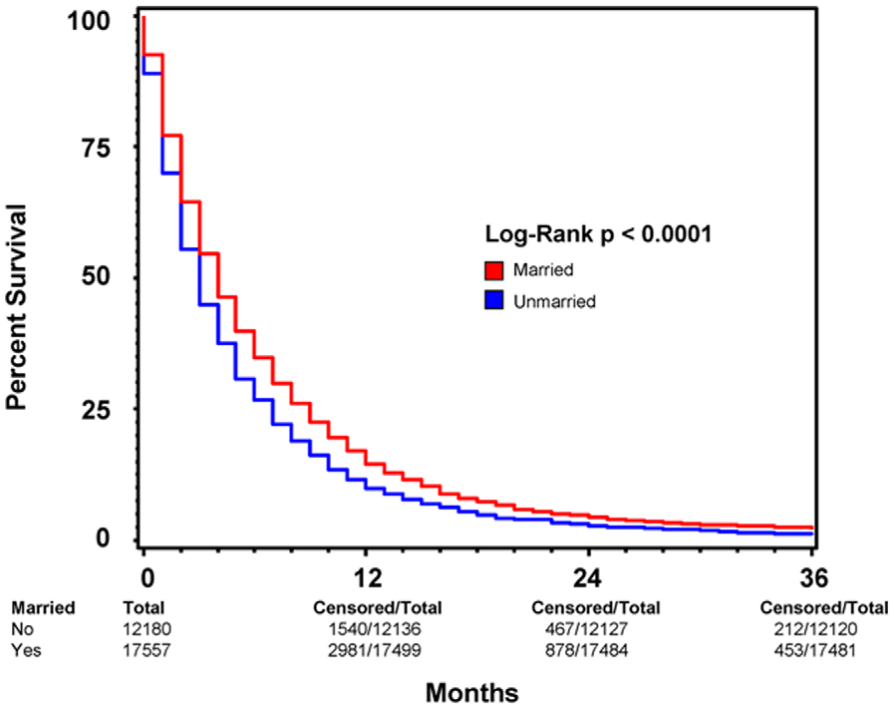
Kaplan-Meier Survival Curves Comparing Patient Marital Status among patients who did not undergo cancer-directed surgery. A Kaplan-Meier survival curve comparing survival based on marital status among all patients diagnosed with pancreatic ductal adenocarcinoma (PC) between 1998 and 2003 with known marital status who *did not undergo* cancer-directed surgery. This comparison illustrates that the increased survival among married patients as compared to those who were non-married at the time of diagnosis continues to be significant regardless of if the patient was not surgically treated for his or her primary tumor. RED indicates patients who were married at the time of diagnosis and BLUE represents patients who were not married at the time of diagnosis. Total patients for each patient group, as well as total and censored patients at 12, 24, and 36 months are indicated. For each time point, “total” indicates the number of patients with ample follow-up information to determine survival at the respective time point. “Censored” indicates the number of patients who lived beyond the respective time point. This method of data representation was chosen rather than indicating at-risk patients for each time point to allow for easier interpretation of overall survival at 12, 24, and 36 months. Total patient number indicated at 0 months is equivalent to total at-risk for each patient group. For each time point, “at-risk” can be considered the number of “censored” patients from the previous time point minus the number of patients lost to follow-up between the previous and current time points. Patients lost to follow-up between time points are indicated by the respective decrease in “total” values.

**Figure 4:**
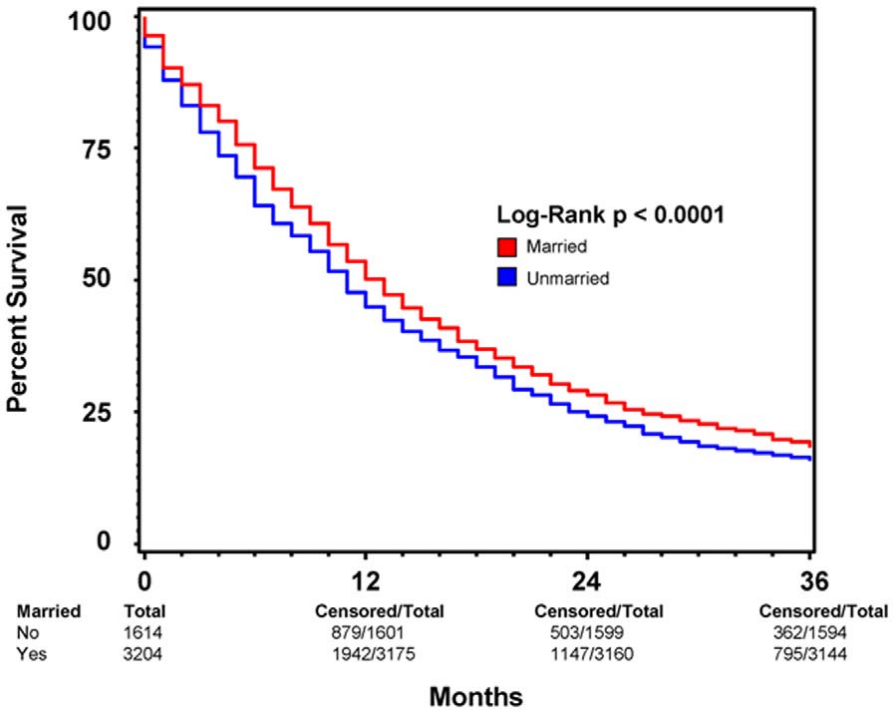
Kaplan-Meier Survival Curves Comparing Patient Marital Status among patients who underwent cancer-directed surgery. A Kaplan-Meier survival curve comparing survival based on marital status among all patients diagnosed with pancreatic ductal adenocarcinoma (PC) between 1998 and 2003 with known marital status who *underwent* cancer-directed surgery. This comparison illustrates that the pro-survival effects of marriage hold true among patients who underwent tumor resection, a procedure that is considered the greatest prognostic factor among patients diagnosed with pancreatic cancer. RED indicates patients who were married at the time of diagnosis and BLUE represents patients who were not married at the time of diagnosis. Total patients for each patient group, as well as total and censored patients at 12, 24, and 36 months are indicated. For each time point, “total” indicates the number of patients with ample follow-up information to determine survival at the respective time point. “Censored” indicates the number of patients who lived beyond the respective time point. This method of data representation was chosen rather than indicating at-risk patients for each time point to allow for easier interpretation of overall survival at 12, 24, and 36 months. Total patient number indicated at 0 months is equivalent to total at-risk for each patient group. For each time point, “at-risk” can be considered the number of “censored” patients from the previous time point minus the number of patients lost to follow-up between the previous and current time points. Patients lost to follow-up between time points are indicated by the respective decrease in “total” values.

Subgroup analysis were performed to determine the effect of marriage on 3-year overall survival in patients with and without cancer-directed surgery. Figures 3 and 4 show Kaplan-Meier survival by marriage in patients without and with cancer-directed surgery, respectively. For patients with cancer-directed surgery, the median survival and 3-year overall survival were 16 (95% CI: 15–17) months and 25% respectively for the married group, compared with 13 (95% CI: 12–14) months and 22% for the unmarried group (p<0.0005). In contrast, for patients without cancer-directed surgery, the median survival and 3-year survival were 4 (95% CI: 3.9–4.1) months and 2% respectively for the married group, compared with 3 (95% CI: 2.9–3.1) months and 1.8% for the unmarried group (p<0.0001). On multivariate analysis of patients who did not undergo cancer-directed surgery, positive marital status as well as female sex, non-African American race, younger age at diagnosis, later year of diagnosis, radiation therapy, and lower stage all remained positive predictors of survival (Table 3). For patients who did undergo cancer-directed surgery, the same positive predictors of 3-year overall survival exist (Table 4).

**Table 3:** Multivariate Analysis of Marital Status on Overall 2 Month and 3 year Survival in Patients with Pancreatic Ductal Adenocarcinoma Without Cancer-Directed Surgery.

	2 Month Survival			3 Year Survival		
Variable	HR (95%CI)		P	HR (95%CI)		P
Marital Status						
Unmarried	1.00			1.00		
Married	0.85	(0.83–0.87)	<0.0001	0.87	(0.85–0.89)	<0.0001
Sex						
Men	1.00			1.00		
Women	0.90	(0.87–0.92)	<0.0001	0.91	(0.89–0.94)	<0.0001
Race						
White	1.00			1.00		
Black	1.12	(1.08–1.16)	<0.0001	1.11	(1.07–1.15)	<0.0001
Other* ≠	0.99	(0.94–1.04)	0.6529	0.98	(0.94–1.03)	0.3772
Age at Diagnosis						
<60	1.00			1.00		
60–74	1.27	(1.23–1.32)	<0.0001	1.27	(1.23–1.31)	<0.0001
>74	1.62	(1.57–1.68)	<0.0001	1.59	(1.54–1.64)	<0.0001
Year of Diagnosis						
2001–2003	1.00			1.00		
1998–2000	1.05	(1.01–1.08)	0.0116	1.04	(1.00–1.07)	0.0299
1993–1997	1.14	(1.12–1.18)	<0.0001	1.12	(1.08–1.15)	<0.0001
1988–1992	1.21	(1.17–1.26)	<0.0001	1.18	(1.14–1.22)	<0.0001
Radiation Therapy						
None	1.00			1.00		
EBRT	0.60	(0.58–0.62)	<0.0001	0.69	(0.67–0.71)	<0.0001
Stage						
Localized	1.00			1.00		
Regional*	1.02	(0.96–1.09)	0.4521	1.08	(1.02–1.13)	0.0065
Distant	1.74	(1.65–1.84)	<0.0001	1.66	(1.58–1.74)	<0.0001

HR indicates hazard ratio; CI indicates confidence interval; EBRT, external beam radiation therapy; A race of Other indicates Asian, or Asian-Pacific Islander.

*Variable shown to not be predictive of 2 month survival.

≠Variable shown to not be predictive of 3 year survival.

**Table 4:** Multivariate Analysis of Marital Status on Overall 2 Month and 3 year Survival in Patients with Pancreatic Ductal Adenocarcinoma With Cancer-Directed Surgery.

	2 Month Survival			3 Year Survival		
Variable	HR (95%CI)		P	HR (95%CI)		P
Marital Status						
Unmarried	1.00			1.00		
Married	0.84	(0.77–0.93)	0.0005	0.90	(0.84–0.97)	0.0047
Sex						
Men	1.00			1.00		
Women	0.86	(0.79–0.95)	0.0015	0.92	(0.86–0.99)	0.0204
Race						
White	1.00			1.00		
Black	1.27	(1.10–1.46)	0.0012	1.21	(1.09–1.36)	0.0006
Other≠	1.26	(1.06–1.49)	0.0088	1.08	(0.95–1.24)	0.2256
Age at Diagnosis						
<60	1.00			1.00		
60–74	1.63	(1.46–1.82)	<0.0001	1.44	(1.33–1.56)	<0.0001
>74	2.06	(1.81–2.35)	<0.0001	1.81	(1.64–1.99)	<0.0001
Year of Diagnosis						
2001–2003	1.00			1.00		
1998–2000	1.14	(1.01–1.29)	0.03	1.16	(1.06–1.27)	0.0009
1993–1997	1.30	(1.16–1.46)	<0.0001	1.25	(1.15–1.36)	<0.0001
1988–1992	1.35	(1.19–1.54)	<0.0001	1.30	(1.17–1.43)	<0.0001
Radiation Therapy						
None	1.00			1.00		
EBRT	0.52	(0.47–0.58)	<0.0001	0.85	(0.79–0.91)	<0.0001
Stage						
Localized	1.00			1.00		
Regional	1.94	(1.67–2.25)	<0.0001	1.77	(1.60–1.96)	<0.0001
Distant	2.91	(2.44–3.46	<0.0001	2.21	(1.95–2.51)	<0.0001

HR indicates hazard ratio; CI indicates confidence interval; EBRT, external beam radiation therapy; A race of Other indicates Asian, or Asian-Pacific Islander.

≠ Variable shown to not be predictive of 3 year survival.

### Perioperative mortality by marriage

The perioperative mortalities at 2 months post diagnosis were 9.5% and 12% among married and unmarried groups respectively (p<0.0005). Factors that correlated with decreased 2-month overall survival on multivariate analysis included being male, African American race, increased age at diagnosis, earlier year of diagnosis, lack of cancer-directed surgery, lack of radiation therapy, more advanced stage, and being unmarried (see Table 5). Marriage yielded a 15% decrease in risk of death at perioperative period.

**Table 5:** Univariate and Multivariate Analysis of Overall 2 month Survival in Patients with Pancreatic Ductal Adenocarcinoma.

	Univariate analysis			Multivariate analysis		
Variable	HR (95%CI)		P	HR (95%CI)		P
Marital Status						
Unmarried	1.00			1.00		
Married	0.81	(0.79–0.83)	<0.0001	0.85	(0.83–0.87)	<0.0001
Sex						
Men	1.00			1.00		
Women	0.96	(0.94–0.98)	0.0005	0.89	(0.87–0.91)	<0.0001
Race						
White	1.00			1.00		
Black	1.14	(1.10–1.18)	<0.0001	1.13	(1.09–1.17)	<0.0001
Other* ≠	1.00	(0.96–1.05)	0.90	1.00	(0.96–1.05)	0.8782
Age at Diagnosis						
<60	1.00			1.00		
60–74	1.29	(1.25–1.33)	<0.0001	1.30	(1.26–1.35)	<0.0001
>74	1.72	(1.66–1.77)	<0.0001	1.67	(1.61–1.72)	<0.0001
Year of Diagnosis						
2001–2003	1.00			1.00		
1998–2000	1.05	(1.02–1.08)	0.0042	1.05	(1.02–1.09)	0.0026
1993–1997	1.13	(1.10–1.17)	<0.0001	1.15	(1.12–1.20)	<0.0001
1988–1992	1.18	(1.14–1.22)	<0.0001	1.23	(1.19–1.27)	<0.0001
Cancer Directed Surgery						
None	1.00			1.00		
Surgery	0.29	(0.27–0.30)	<0.0001	0.43	(0.41–0.45)	<0.0001
Radiation Therapy						
None	1.00			1.00		
EBRT	0.44	(0.43–0.46)	<0.0001	0.59	(0.57–0.61)	<0.0001
Stage						
Localized	1.00			1.00		
Regional*	1.03	(0.97–1.08)	0.36	1.13	(1.07–1.20)	<0.0001
Distant	2.32	(2.20–2.44)	<0.0001	1.89	(1.79–1.99)	<0.0001

HR indicates hazard ratio; CI indicates confidence interval; EBRT, external beam radiation therapy; A race of Other indicates Asian, or Asian-Pacific Islander.

*Variable shown to not be predictive of survival in univariate analysis.

≠Variable shown to not be predictive of survival in multivariate analysis.

### Comparing the effect of marriage on 2-month and 3-year survival

Interestingly, while positive marital status remained a predictor for increased short-term and long-term survival in both patients who underwent cancer-directed surgery and those who did not, the amount by which it positively predicts survival is variable between the two groups. As is illustrated in Figures 3 and 4, the amount by which marital status predicts overall survival at 2 months in each of the two groups is very similar (15% for patients without surgery vs. 16% for those with surgery), though increased variability is present among patients who underwent cancer-directed surgery as can be seen by the wider range of the 95% CI (Tables 3 and 4). The ability of marital status to predict overall survival 3 years post diagnosis, however, is greater in patients who did not undergo cancer directed surgery, having a HR of 0.87 vs. 0.90 for patients with cancer-directed surgery. This difference may reflect that the ability of marital status to predict long-term survival is enhanced with more advanced stage disease which would preclude cancer-directed surgery. Conversely, this finding may also signal that, though marriage is a positive predictor of overall survival, cancer-directed surgery may be such a significant predictor of survival that its effects partially drown-out the predictive ability of marital status.

## Discussion

PDAC is the deadliest cancer in the world, with an overall 5-year survival rate of 5% [2]. Currently, no current treatments result in objective clinical response in more than 20% of patients and no treatment regimen has been found that extends survival beyond a few months [6]. Thus, all avenues that can be found to increase survival in afflicted patients are of significant clinical impact. This study is the first to epidemiologically link marital status with survival in PDAC in the American population. While traditional prognostic factors such as age, stage, tumor resection, and tumor irradiation had greater impacts on patient survival, we observed that patient marital status was a more significant predictor of both short-term and long-term survival than patient sex or race, two factors well established to correlate with post-diagnostic life-span in PDAC. Additionally, marital status had a greater impact on peri-operative survival than did a diagnosis of regional tumor involvement versus localized disease. Such impact of patient marital status was found to be independent of patient surgical status as well, precluding any confounding impact that may have resulted from such treatment.

Marriage is well known to have a positive impact on patient survival across many malignancies though its effects, both in quantity and quality, vary from study to study and between cancers. The impact of marriage on cancer survival has been studied using both SEER as well as other country-specific cancer databases. Currently the most comprehensive (with regard to cancer type) study yet undertaken linking marital status with cancer prognosis, and the only study to date including pancreatic cancer in its analysis, looked at malignancies of the stomach, colon/rectum, lung, breast, cervix, corpus uteri, ovary, prostate, pancreas, and bladder as well as two hematologic malignancies (multiple myeloma and leukemia) using combined data from the Norwegian Cancer Registry and Norwegian Population Register and Population Census from 1960, 1970 and 1980 [4]. This study found that marriage increased 5-year relative survival by 15% in cancer in general, with the greatest increase in survival observed in males with malignant melanoma (Hazard Ratio (H.R.): 1.56, p<0.05) and the least in leukemia and uterine cancer. In total, marriage was found to be a statistically significant independent predictor of survival in stomach (H.R.: 1.09–1.15), lung (H.R.: 1.13–1.23), and pancreatic cancers (H.R.: 1.03–1.32) among both sexes as well as in females with breast (H.R.: 1.13), ovarian (H.R.: 1.49) and cervical cancers (H.R.: 1.28) and males with prostate cancer (H.R.: 1.12), bladder cancer (H.R.: 1.49), and myeloma (H.R.: 1.56) (p<0.05 for all) [4]. Similar to our results, this Norwegian study found that marriage increases long-term survival in pancreatic cancer by 3–32%, suggesting that our results are consistent across multiple primarily-Caucasian populations. In contrast, however, this Norwegian study did not analyze short-term survival for any cancer, but did separate non-married persons into individual groups based on being never-married, divorced, or widowed as well as analyzing each sex individually. Further, this study analyzed relative survival, not overall survival and compared the survival advantage in cancer patients to that conferred by marriage in the general population, preventing erroneous conclusions from being drawn with regard to the survival advantage of marriage in the specific context of malignancy. Such analysis, however, is only considered necessary upon analyses of non-aggressive cancers with long survival times [4]. Another study was published in 2005 using SEER data to analyze the affect of marital status on survival in women over 65 years of age diagnosed with breast cancer and found that marriage increased 3-year overall-survival by 25% [7]. Interestingly, this study merged the SEER survival, tumor, and treatment data with Medicare data to analyze potential confounding effects of sociodemographics and comorbidities, though neither was found to substantially affect the positive association of marriage with improved survival (though increased income was found to improve the association). Further contrasting this study with ours is the fact that only patients diagnosed between 1991 and 1995 were entered into the analysis and, again, short-term survival was not analyzed [7]. A third study, published just this year, showed that marriage elicited improved 5-year survival in women diagnosed with cervical cancer, however this survival advantage was lost when corrected for tumor stage and cancer-directed radiation treatment, suggesting that marriage itself may increase the likelihood of early diagnosis and/or aggressive treatment of cervical cancer but does not itself independently affect survival [8]. Like ours, this study was conducted using the SEER database however, in contrast, only patients diagnosed from 1992 to 1996 (7997 women in total) were included in the study, non-married persons were further classified into never-married, divorced/separated, and widowed groups, and no short-term survival was analyzed. Beyond these studies directly linking marriage to survival, it has also been found that an increase in social support and self-efficacy, both being linked to marriage, is strongly correlated with decreased rates of depression, distress, and anxiety, all of which have been linked to increased morbidity and mortality in cancer patients [9]–[14].

While the mechanism underlying the improved survival associated with marriage in patients with PDAC is not entirely clear, two potential reasons are proposed: those with psychiatric or physical impairments may be less likely to marry, thereby causing this observation to reflect a healthy selection bias, or marriage may provide with it inherent benefits to health through the support system it establishes [9]. Of note, these two potential mechanisms for increased survival among the married are not mutually exclusive but, for use in understanding and improving prognosis in PDAC, only analysis of the latter is clinically practical. Multiple mechanisms exist by which marriage itself may provide increased survival in pancreatic cancer, most of which pertain to psychosocial support through increased access to healthcare, increased prominence of healthful lifestyles, or a decreased quantity and quality of stressors. As an example, married persons may enjoy greater financial resources than those who are unmarried due to gains from economies of scale. Evidence for this correlation between marriage and finances is widespread, has been found throughout the world, and has the potential to allow for decreased non-medically related stress due to a perceived economic “safety-net” as well as increasing access to healthcare, better food, and better housing [9]. Additionally, studies have repeatedly shown that marriage may positively influence behaviors such as undergoing health screenings, accessing appropriate healthcare resources, improved diet, and routinely partaking in exercise, all of which are factors that promote health and have been shown to prolong life [9], [10], [15]. Evidence for the mechanism behind this comes from a recent study, in which it was found that degree of loneliness and perceived social support have the greatest effect on increasing healthy behaviors. Marriage, too, may provide for earlier detection of symptoms from both the primary disease as well as life-threatening or life-shortening treatment toxicities, allowing them to be addressed more rapidly and thus stemming their effects before they can have a negative impact on survival [10], [15]. Married persons receive better social support as well, giving them a greater sense of happiness and acceptance, often referred to as self-efficacy [9], [16]. This support helps prevent the illness from encompassing the entire life of the patient, allowing them to focus on something joyful and thereby giving the patient a mental outlet associated with fundamental pleasure which may translate to a more concrete desire to live. Further, this social support often coincides with increased practical support such as transportation, paperwork, assistance with activities of daily living, and household chores; reducing day-to-day stress of the patient and enabling them to fully focus on treatment. While this practical support not only increases the ease with which patients can access their physicians, medications, and treatment facilities, all of which have obvious positive impacts on survival, the accomplishment of reduction in stress and the anxiety that comes with it cannot be overlooked. Indeed, none of these aspects of marriage affecting survival must exist inside a vacuum, alone being responsible for increasing survival in PDAC but rather all likely work in conjunction to establish the global qualitative reality that marriage provides a mental and physical safety net for those diagnosed with deadly disease.

While this current study is both enlightening and relevant to clinical practice, it is not without limitations. The lack of detailed patient information, such as patient financial status, the existence of preexisting conditions, and mental health, prevent a subset of potential confounders from being accounted for in our analysis as well as disallowing the ability to propose specific mechanisms by which marriage offers increased survival in PDAC. Additionally, the lack of relevant aspects of patient history prevents the deciphering of whether the positive correlation between marital status and PDAC survival is due to inherent benefits from marriage or is simply a reflection of a healthy selection bias found in married patients. The insufficient inclusion of tumor grade in SEER PDAC patient data also precludes any analysis of tumor morphology as a potential confounder. Further, due to the absence of information on chemotherapy included in the SEER database, its effect on survival could not be evaluated. This analysis also did not separate non-married patients based on being never-married, divorced, or widowed, did not analyze each sex individually, did not take into account cohabitation, and did not account for length of time each patient had been married/non-married at the time of diagnosis. Importantly, as pancreatic cancer-specific survival data is not available in the SEER database, the impact of marital status on pancreatic cancer mortality due to non-cancer related events also could not be assessed. While this study remains the first of its kind analyzing the effects of marital status on survival in PDAC in the American population, each of these limitations much be addressed in future studies to fully decipher what affect marriage truly has on PDAC prognosis.

This investigation of the relationship between marital status and PDAC survival opens up a potential mechanism that may be influencing the survival of patients with the world's deadliest cancer. Discovery of the true cause(s) underlying the ability of marriage to increase survival in pancreatic cancer is necessary. Once these mechanisms are deciphered, the clinical challenge will be to incorporate prognostically beneficial aspects of marriage into the treatment of all patients diagnosed with PDAC, allowing for the establishment of more holistic approaches which may positively influence patient morbidity and mortality. In this way, the positive impact on survival provided by marriage can be enjoyed by all PDAC patients, regardless of marital status.
